# Return of the Coronavirus: 2019-nCoV

**DOI:** 10.3390/v12020135

**Published:** 2020-01-24

**Authors:** Lisa E. Gralinski, Vineet D. Menachery

**Affiliations:** 1 Department of Epidemiology, Gillings School of Global Public Health, University of North Carolina, Chapel Hill, NC 27514, USA; lgralins@email.unc.edu; 2 Department of Microbiology and Immunology, University of Texas Medical Branch, Galveston, TX 77555, USA; 3 Institute for Human Infections and Immunity, University of Texas Medical Branch, Galveston, TX 77555, USA

**Keywords:** 2019-nCoV, novel CoV, Wuhan, Wuhan pneumonia, coronavirus, emerging viruses, SARS-CoV, MERS-CoV

## Abstract

The emergence of a novel coronavirus (2019-nCoV) has awakened the echoes of SARS-CoV from nearly two decades ago. Yet, with technological advances and important lessons gained from previous outbreaks, perhaps the world is better equipped to deal with the most recent emergent group 2B coronavirus.

## 1. Emergence

**Figure 1:**
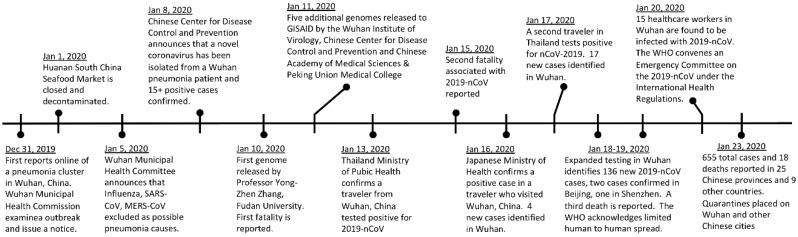
Timeline of the key 2019-nCoV events.

The third zoonotic human coronavirus (CoV) of the century emerged in December 2019, with a cluster of patients with connections to Huanan South China Seafood Market in Wuhan, Hubei Province, China. Similar to severe acute respiratory syndrome coronavirus (SARS-CoV) and Middle East respiratory syndrome coronavirus (MERS-CoV) infections, patients exhibited symptoms of viral pneumonia including fever, difficulty breathing, and bilateral lung infiltration in the most severe cases [1]. News reports of patients with an unknown pneumonia were first identified on 31st December with the Wuhan Municipal Health Commission saying they were monitoring the situation closely (Figure 1). On 1st January 2020, the seafood market was closed and decontaminated while countries with travel links to Wuhan went on high alert for potential travelers with unexplained respiratory disease. After extensive speculation about a causative agent, the Chinese Center for Disease Control and Prevention (CDC) confirmed a report by the Wall Street Journal and announced identification of a novel CoV on 9th January [2]. The novel CoV (2019-nCoV) was isolated from a single patient and subsequently verified in 16 additional patients [3]. While not yet confirmed to induce the viral pneumonia, 2019-nCoV was quickly predicted as the likely causative agent.

The first sequence of 2019-nCoV was posted online one day after its confirmation on behalf of Dr. Yong-Zhen Zhang and scientists at Fudan University, Shanghai [4]. Subsequently, five additional 2019-nCoV sequences were deposited on the GSAID database on 11th January from institutes across China (Chinese CDC, Wuhan Institute of Virology and Chinese Academy of Medical Sciences & Peking Union Medical College) and allowed researchers around the world to begin analyzing the new CoV [5]. By 17th January, there were 62 confirmed cases in China and importantly, three exported cases of infected travelers who were diagnosed in Thailand (2) and Japan (1) [6]. The sequences of these exported cases and several additional 2019-nCoV isolated in China have also been deposited on the GSAID database [5]. Diagnostic tests have subsequently been developed and some are being used on suspect cases identified in other locations including Vietnam, Singapore, and Hong Kong [7]. To date there have been twenty-six fatalities associated with 2019-nCoV infection, many of these cases had significant co-morbidities and were older in age (>50). A range of disease has been observed highlighted by fever, dry cough, shortness of breath, and leukopenia; patients have included mild cases needing supportive care to severe cases requiring extracorporeal membrane oxygenation; however, compared to SARS-CoV (10% mortality) and MERS-CoV (35% mortality), the 2019-nCoV appears to be less virulent at this point with the exception of the elderly and those with underlying health conditions. Initial monitoring of case close contacts had not revealed any further 2019-nCoV cases. However, modeling analysis based on official case numbers and international spread suggested that there may be cases going undetected [8]. On 19th January, these fears were seemingly confirmed as an additional 136 cases were added from further surveys raising the total in Wuhan to 198 infected patients [9]. Among the 198 total cases in Wuhan, 170 remained in hospitals, 126 mostly with mild symptoms, 35 in serious condition, and 9 in critical condition. The expanded numbers and extended range of onset dates (12 December 2019–18 January 2020) suggested likely human to human transmission or ongoing transmission from a market or other primary sources. On 20th January, the outbreak was further expanded to other parts of China (Beijing, Shanghai, & Shenzhen) as well as another exported cases to South Korea. As of January 24, the total case number has expanded to at least 870 total cases with 26 deaths across 25 provinces in China and 19 exported cases in 10 countries [10]. Public health authorities have quarantined travel from Wuhan to limit the spread of the virus and reports indicate other Chinese cities have also been isolated [11]. With the heavy travel season for lunar New Year underway in Asia, major concerns exist for the 2019-nCoV outbreak to continue and spread.

## 2. Origins of 2019-nCoV

The source of the 2019-nCoV is still unknown, although the initial cases have been associated with the Huanan South China Seafood Market. While many of the early patients worked in or visited the market, none of the exported cases had contact with the market, suggesting either human to human transmission or a more widespread animal source [6]. In addition to seafood, it is reported on social media that snakes, birds and other small mammals including marmots and bats were sold at the Huanan South China Seafood Market. The WHO reported that environmental samples taken from the marketplace have come back positive for the novel coronavirus, but no specific animal association has been identified [6]. An initial report suggested that snakes might be the possible source based on codon usage [12], but the assertion has been disputed by others [13]. Researchers are currently working to identify the source of 2019-nCoV including possible intermediate animal vectors.

A zoonotic reservoir harkens back to the emergence of both SARS- and MERS-CoV. SARS-CoV, the first highly pathogenic human CoV, emerged in 2002 with transmission from animals to humans occurring in wet markets. Surveillance efforts found SARS-CoV viral RNA in both palm civets and raccoon dogs sold in these wet markets [14]; however, SARS-CoV was not found in the wild, suggesting that those species served as intermediary reservoir as the virus adapted to more efficiently infect humans. Further surveillance efforts identified highly related CoVs in bat species [15]. More recent work has demonstrated that several bat CoVs are capable of infecting human cells without a need for intermediate adaptation [16,17]. Additionally, human serology data shows recognition of bat CoV proteins and indicates that low-level zoonotic transmission of SARS-like bat coronaviruses occurs outside of recognized outbreaks [18]. MERS-CoV is also a zoonotic virus with possible origins in bats [19,20], although camels are endemically infected and camel contact is frequently reported during primary MERS-CoV cases [21]. For SARS-CoV, strict quarantine and the culling of live markets in SE Asia played a major role in ending the outbreak. With the cultural importance of camels, a similar approach for MERS-CoV was not an option and periodic outbreaks continue in the Middle East. These lessons from SARS and MERS highlight the importance of rapidly finding the source for 2019-nCoV in order to stem the ongoing outbreak.

## 3. Susceptible Populations

With limited patient data, it is difficult to make robust declarations about populations that may be most susceptible to 2019-nCoV. However, disease severity following SARS- and MERS-CoV corresponded strongly to underlying host conditions including age, biological sex, and overall health [22]. Early patient reports from 2019-nCoV find similar trends. Severe illness with 2019-nCoV has been associated with elderly patients (>60 years old), including twenty-six lethal cases. These findings correspond to increased severity and death in people over the age of 50 following both SARS and MERS-CoV infection [23,24]. Similarly, the underlying health of the patient likely plays a critical role in overall susceptibility. For the 2019-nCoV, limited comorbidity data is available; however, the twenty-six patients that have succumbed to the novel CoV had significant health conditions including hypertension, diabetes, heart and/or kidney function issues that may have made them more susceptible. For the MERS-CoV outbreak, smoking, hypertension, diabetes, cardiovascular disease, and/or other chronic illnesses have been present in the majority of deaths and correspond to findings in animal models [25]. The results indicate vigilance is necessary for these vulnerable patients following 2019-nCoV infection.

## 4. Insights from the 2019-nCoV Sequence

The rapid sequencing of the nearly 30,000 nucleotide 2019-nCoV genome by Dr. Zhang’s group at Fudan University and several other groups in China illustrate the dedication and increased capacity of the scientific infrastructure in China [4,5]. For SARS-CoV, the causative agent was unknown for months and subsequently took over four weeks until a full genome was released [26]. Similarly, MERS-CoV was only identified after several months of testing and a full-length genome available about a month later [27]. In contrast, time from the first date of patient onset (12 December 2019) to the report of several 2019-nCoV full-length genomes took less than one month. Combined with the immense pressure of an ongoing outbreak with an unknown agent, the effort of these scientists should be considered nothing less than remarkable. 

Building from the sequence, the nucleotide alignment quickly distinguished the novel virus as a group 2B CoV, distinct from the SARS-CoV strains [4,5]. Examining the whole genome, 2019-nCoV maintains ~80% nucleotide identity to the original SARS epidemic viruses. Its closest whole genome relatives are two bat SARS-like CoVs (ZC45 and ZXC21) that shared ~89% sequence identity with 2019-nCoV; these CoV sequences were deposited in early 2018 from Zhejiang province in *R. sinicus* bats in China. Comparing across the deposited 2019-nCoV strains finds > 99.5% conservation; the lack of diversity suggests a common lineage and source with emergence not likely having occurred that long ago [28,29]. A recent report has subsequently identified a bat CoV sequence, RaTG3, with 92% sequence identity with the novel virus which argues for bat origins for the 2019-nCoV [30].

We next shifted analysis to the nucleocapsid (N) protein, the most abundant protein produced in CoVs. Generally, the N protein is well conserved across CoV families including group 2B [31]. The N protein for 2019-nCoV is no exception with ~90% amino acid identity to the SARS-CoV N protein. While less conserved than other group 2B CoVs like HKU3-CoV and SHC014-CoV, 2019-nCoV antibodies against the N protein would likely recognize and bind the SARS-CoV N protein as well. N antibodies do not provide immunity to 2019-nCoV infection, but the cross reactivity with SARS-CoV N protein would allow a serum based assay to determine exposure to the novel CoV in asymptomatic cases. While previous studies have found serum reactivity to group 2B virus N proteins in Chinese populations [18], exposure to 2019-nCoV should increase the dilution factor substantially if exposure/infection had occurred. Importantly, this information may provide insights about susceptibly and potential routes of spread through asymptomatic carriers.

Examining further, we next compared the spike proteins, the critical glycoprotein responsible for virus binding and entry. Overall, the 2019-nCoV spike protein has roughly 75% amino acid identity with SARS-CoV, which is less conserved than other group 2B CoVs including HKU3-CoV [31]. However, narrowing analysis to the spike receptor binding domain (RBD) of SARS-CoV (amino acids 318–518), the 2019-nCoV RBD is 73% conserved relative to the epidemic RBD. This conservation level places the 2019-nCoV RBD between HKU3-4 (62.7% conservation), a bat virus that cannot use human ACE2, and rSHC014 (80.8%), the most divergent bat CoV spike known to use human ACE2 for entry [16,32]. Importantly, the key binding residues for SARS-CoV have been identified [33]; among these fourteen residues predicted to interact directly with human ACE2, the receptor for SARS-CoV, eight amino acids are conserved in 2019-nCoV. Notably, several of these residues are also conserved relative to WIV1- and WIV16-CoV, two bat strains closely related to SARS-CoV and known to use human ACE2 [17,34]. Initial structural modeling suggest that the 2019-nCoV may be able to use human ACE2 as a receptor, although its affinity m be reduced relative to the epidemic SARS-CoV strains [35]. A subsequent report demonstrated that the receptor binding domain of 2019-nCoV was capable of binding ACE2 in the context of the SARS-CoV spike protein [36]. In addition, another rapid report links demonstrates 2019-nCoV uses ACE2 receptors from human, bat, civets, and swine [30]. Together, the modeling, pseudotyping, and infection data provide strong evidence for human ACE2 being the receptor for 2019-nCoV. 

## 5. Achieving Koch Postulates

Traditional identification of a microbe as the causative agent of disease requires fulfillment of Koch’s postulates, modified by Rivers for viral diseases [37]. At the present time, the 2019-nCoV has been isolated from patients, detected by specific assays in patients, and cultured in host cells (one available sequence is identified as a passage isolate), starting to fulfill these criteria. Given the recentness of the 2019-nCoV outbreak, at this point there is no animal model available to fulfill the remaining criteria: 1) testing the capability of 2019-nCoV to cause respiratory disease in a related species, 2) re-isolating the virus from the experimentally infected animal and 3) detection of a specific immune response. These efforts will surely be an area of intense research in the coming months both in China and in CoV research laboratories around the world. 

Notably, generating small animal models of coronavirus disease can be difficult. While SARS-CoV readily infected laboratory mice, it does not cause significant disease unless the virus is passaged to adapt to the mouse host [38]. Infection of primates produces a more mild disease than that observed in humans, although fever and pulmonary inflammation were noted [39,40]. MERS-CoV is incapable of infecting rodent cells without engineering changes in critical residues of the receptor protein, DPP4 [41,42]. However, MERS-CoV does infect non-human primates [43]. As such, MERS mouse models of disease required a great deal of time to develop and are limited in the types of manipulations that can be performed [41]. At this point, the infectious capability of the 2019-nCoV for different species and different cell types is unknown. Early reports suggest that the virus can utilize human, bat, swine, and civet ACE2 [30]; notably, the group found mouse Ace2 was not permissive for 2019-nCoV infection Dissemination of virus stocks and/or de novo generation of the virus through reverse genetics systems will enable this research allowing for animal testing and subsequent completion of Koch’s postulates for the new virus.

## 6. Threat for Spread: Human to Human, Health Care Workers, and Super Spreaders

While the Huanan seafood market in Wuhan has been associated with the majority of cases, many of the recent cases do not have a direct connection [9]. This fact suggests a secondary source of infection, either human to human transmission or possibly infected animals in another market in Wuhan. Both possibilities represent major concerns and indicate the outbreak has the potential to expand rapidly. For human to human transmission, there was limited data in the initial set of cases; one family cluster is of three men who all work in the market. Similarly, a husband and wife are among the patients, with the wife claiming no contact with the market. In these cases, direct human to human infection may have been possible; alternatively, a contaminated fomite from the market may also be responsible as surfaces all around the market were found to test positive 2019-nCoV. However, the major increase in the number of cases, the lack of direct connection to the Wuhan market for many cases, and the infection of health care works all suggest human to human spread is likely [9,44]. Importantly, until the source of the virus is found, it will be difficult to distinguish zoonotic versus human to human spread.

In the early part of the outbreak, the absence of infection in health care workers argued for inefficient human to human spread and distinguished 2019-nCoV from both SARS-CoV and MERS-CoV. In the two prior CoV epidemics, health care settings served as a major transmission point fueling both outbreaks. Based on WHO data, 1 in 10 MERS-CoV cases have been found to be health care workers; these patients generally have reduced disease and death likely due to younger age and absence of existing health conditions. The recent reports of numerous infected health care workers in Wuhan indicate human to human infection can occur with 2019-nCoV and may be the product of a super spreading patient [44]. However, while large swaths of healthcare workers are not getting sick as seen with SARS and MERS-CoV, it may be too early to rule out their potential exposure to the novel CoV as their disease may be asymptomatic. While not described during the SARS-CoV outbreak, asymptomatic cases ranged from 12.5% to 25% in some MERS-CoV studies [45]. A similar phenomenon may be occurring with 2019-nCoV and would make stopping the outbreak even more difficult to contain. 

Another parameter to consider is the possibility of super spreading in the context of 2019-nCoV. Super spreading is the amplified transmission of a virus by individuals in a population and has been suggested by at least one news report [44]. Both SARS- and MERS-CoV outbreaks had documented evidence of super spreading patients [46]. In general, both epidemic CoVs maintain a low R_0_, the rate spread from an individual infected patient. However, roughly 10% of SARS- and MERS-CoV patients have been associated with super spreading and an R_0_ > 10. These cases seeded a significant portion of the epidemic around the world. Notably, neither mutations in the viruses nor severity of disease were found to be associated with super spreading, implying that host factors contribute to the phenotype [47]. For 2019-nCoV, contact tracing to date suggest limited human to human spread and a low R_0_. However, the recent increase in cases, both in and outside Wuhan could signal the existence of super-spreading individuals fueling the outbreak. Alternatively, super spreading could occur from the zoonotic source which has been seen in other disease outbreaks [10]. In any event, the possibility of super spreading may continue to play a role in this ongoing 2019-nCoV outbreak.

## 7. Emerging Diseases in the Age of Social Media

News of the 2019-nCoV came to widespread attention through the internet. Over the years, websites like FluTrackers.com, ProMED (promedmail.org), and others have permitted the collection of disease information from around the world and facilitated dissemination to interested parties. In 2012, MERS-CoV first drew attention as a “novel coronavirus” entioned on ProMED Mail and subsequently through conversation on twitter between science journalists, virologists, and public health experts. Eight years later, a more connected network quickly dissected statements from the Wuhan Municipal Health Commission and speculated about possible causes. Early during an outbreak, it can be difficult to distinguish between rumors with elements of truth versus baseless fear mongering. This fact can be exacerbated by language barriers and off the record sources. However, in this case, speculation of a novel coronavirus was fed by carefully worded statements that specifically excluding some virus families (influenza, adenovirus), but only excluded SARS-CoV and MERS-CoV for coronaviruses. Coupled with memories of the SARS outbreak, many worried that the truth may be held back. When the agent was finally confirmed as a CoV, the world acted with both worry and relief: the outbreak would not be hidden. 

While far from perfect, the government response to 2019-nCoV provides a stark contrast to the SARS outbreak at the beginning of the century. The rapid release of 2019-nCoV sequences permitted the research community to quickly become engaged, providing analysis and developing diagnostic tests. Both the Chinese CDC and the Wuhan Municipal Health Commission have posted regular updates of confirmed case numbers and patient statuses enabling public health authorities to monitor the situation in real time. Researchers from around the world have connected on social media to compare updated sequence information and highlight key unknowns about the outbreak. While not always provided in a timely manner, the ability to share news updates and data in real time with researchers and public health officials around the world signals a major change in the response to outbreaks. This connectivity has facilitated awareness as well as new collaborations and a rapid response by the global research community. While there are many unknowns with 2019-nCoV, the world is engaged and prepared to battle the newest emergent virus strain. Perhaps this means the lessons from the SARS outbreak have truly been learned.
